# The interaction between dietary habits, BDNF, CCK, and gut microbiota in the pathophysiology of Alzheimer’s disease

**DOI:** 10.1002/alz.090041

**Published:** 2025-01-09

**Authors:** Caitlin E Montgomery, Jessica Arango Hipsley, Olivia Schuh, Anjali Shah, Deepesh Khanna

**Affiliations:** ^1^ Nove Southeastern Dr. Kiran C. Patel College of Osteopathic Medicine, Clearwater, FL USA; ^2^ Nova Southeastern Kiran C. Patel College of Osteopathic Medicine, Clearwater, FL USA; ^3^ Dr. Kiran C. Patel College of Osteopathic Medicine ‐ TBR, Clearwater, FL USA; ^4^ Nova Southeastern Kiran C. Patel College of Osteaopathic Medicine, Clearwater, FL USA; ^5^ Nova Southeastern Dr. Kiran C. Patel College of Osteopathic Medicine ‐ TBR, Clearwater, FL USA

## Abstract

**Background:**

Research heavily suggests that brain‐derived neurotrophic factor (BDNF), vital for neuronal growth and plasticity, and cholecystokinin (CCK), a satiety hormone that regulates BDNF levels, are altered in Alzheimer’s Disease pathophysiology. Factors such as dysbiosis of gut microbiota and poor food habits may affect CCK and BDNF release and brain function. The objective is to evaluate the effects of dietary habits, gut microbiota, and exercise on BDNF and CCK release in Alzheimer’s Disease patients.

**Methods:**

A comprehensive review on how BDNF and CCK levels are affected in Alzheimer’s patients was conducted using databases like PubMed and Google Scholar. Search string keywords included “Alzheimer’s,” “BDNF,” “CCK,” paired with “exercise,” “diet,” and “gut microbiota.” The inclusion criteria included original articles published from 2012‐2024 from peer‐reviewed journals and studies exploring CCK and BDNF interaction with diet, exercise, and gut microbiota. Eligible studies were excluded if BDNF and CCK levels were associated with diseases other than Alzheimer’s. Papers were not restricted to the US population.

**Results:**

Literature suggests BDNF levels significantly increase with exercise in Alzheimer’s disease patients. BDNF levels decrease in mice fed a high‐fat diet that reflects the Western diet. Research on the connection between gut microbiota and Alzheimer’s disease has also been explored in human and mice studies. A study conducted on human participants showed serum BDNF levels to significantly increase at Week 12 in the probiotic’s treatment group in contrast to the placebo group (−3.32 ± 2.35 ng/mL). A study on healthy mice that were given fecal microbiota transplantation from 5XFAD mice with major features of Alzheimer’s Disease amyloid pathology demonstrated elevated proinflammatory cytokines and decreased neurogenesis in the brain. Studies exploring CCK levels found that higher CCK was significantly associated with greater gray matter volume in areas of the brain that play a role in memory primarily spanning cingulate cortex and parahippocampal gyrus. In Alzheimer’s patients, CCK levels were found to be significantly lower.

**Conclusion:**

Findings collectively suggest the complex interactions between diet, exercise, and neurocognitive outcomes, with both dietary interventions and physical activity, positively influence synaptic plasticity, cognitive performance, and biomarkers associated with neurodegenerative diseases.

